# Rational design of adhesives for effective underwater bonding

**DOI:** 10.3389/fchem.2022.1007212

**Published:** 2022-10-31

**Authors:** Sidi Li, Chuao Ma, Bin Hou, Hongliang Liu

**Affiliations:** ^1^ College of Chemistry and Chemical Engineering, Yantai University, Yantai, Shandong, China; ^2^ Shandong Laboratory of Yantai Advanced Materials and Green Manufacturing, Yantai, Shandong, China

**Keywords:** underwater bonding, underwater adhesives, interfacial water, interfacial adhesion, cohesiveness

## Abstract

Underwater adhesives hold great promises in our daily life, biomedical fields and industrial engineering. Appropriate underwater bonding can reduce the huge cost from removing the target substance from water, and greatly lift working efficiency. However, different from bonding in air, underwater bonding is quite challenging. The existence of interfacial water prevents the intimate contact between the adhesives and the submerged surfaces, and water environment makes it difficult to achieve high cohesiveness. Even so, in recent years, various underwater adhesives with macroscopic adhesion abilities were emerged. These smart adhesives can ingeniously remove the interfacial water, and enhance cohesion by utilizing their special physicochemical properties or functional groups. In this mini review, we first give a detail introduction of the difficulties in underwater bonding. Further, we overview the recent strategies that are used to construct underwater adhesives, with the emphasis on how to overcome the difficulties of interfacial water and achieve high cohesiveness underwater. In addition, future perspectives of underwater adhesives from the view of practical applications are also discussed. We believe the review will provide inspirations for the discovery of new strategies to overcome the obstacles in underwater bonding, and therefore may contribute to designing effective underwater adhesives.

## Introduction

Underwater bonding is highly demand in wide range of areas (Cui et al., 2017; Fan and Gong, 2021; Wang Z. M., et al, 2021; Wu J. et al., 2022). For example, in our daily life and industrial field, it often requires to directly repair the water pipeline leakage, attach the underwater sensor, or even repair the broken hull in water. In medical applications, doctors usually need to seal the wounds in moisture environment or even under blood. Efficient underwater bonding can greatly simplify the working procedures with no need for creating dry surfaces, thereby lifting working efficiency and reducing cost (Xia et al., 2021). However, achieving underwater bonding is commonly challenging (Ahn et al., 2015; Narayanan et al., 2021). And commercial man-made adhesives, such as cyanoacrylate (Super glue), vinyl acetate (Elmer’s glue), as well as most epoxy and polyurethane glue, cannot perform well in underwater adhesion (Li et al., 2017; White and Wilker, 2011; North et al., 2017; Cheng et al., 2022).

The challenges in underwater bonding mainly result from the interfacial water on the submerged surfaces, and the difficulties in achieving high cohesiveness underwater (Kamino, 2008; Kamino, 2013; Waite, 2017; Fan and Gong, 2021; Narayanan et al., 2021; Cheng et al., 2022). In recent years, researchers have proposed various strategies to overcome these obstacles, and therefore developed new types of adhesives that were capable of underwater bonding. In this mini review, we first introduced the difficulties in underwater bonding, and then overviewed the main discovered strategies that are used to realize underwater macroscopical adhesion. In this part, we mainly focused on the strategies of creating underwater adhesives with macroscopical underwater bonding capacities. In the last part, the future perspectives of adhesives according to practical applications were discussed.

## Obstacles in underwater bonding

**FIGURE 1:**
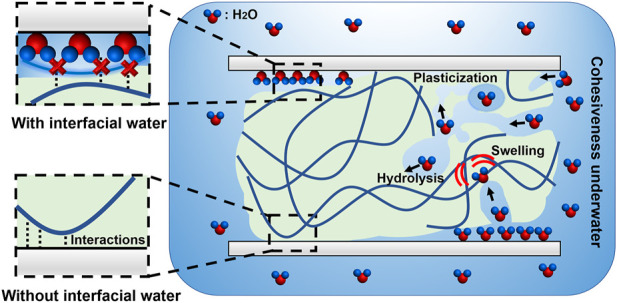
Obstacles and difficulties in underwater bonding. Underwater bonding faces with the obstacles of interfacial water and the difficulties in achieving high cohesiveness underwater. Without the interfacial water, the adhesive molecules are able to bond with the target surfaces by multiple predetermined interactions. However, the interfacial water hinders the intimate contact between the adhesive molecules and surfaces, causing the failure of the underwater bonding. In addition, achieving high cohesiveness underwater is also challenging. The adhesives face the difficulties in curing underwater and the risk of hydrolysis, swelling, plasticization in water, which may weaken the structure of the adhesives.

Water and adhesives are in conflict (Akdogan et al., 2014; Myslicki et al., 2020), and achieving efficient underwater bonding is challenging (Figure 1). One reason is that interfacial water disrupts the adhesion of the adhesives on submerged surfaces (Petrone et al., 2015; Narayanan et al., 2021). Especially for hydrophilic submerged surfaces, such as most minerals, metals, oxides, some fabrics and biological surfaces (Rapp et al., 2016), water molecules and salts (if any) can strongly bind to the surfaces and form hydration layers, thereby preventing intimate contact between the adhesives and surfaces. For example, in saline solution, a hydration layer with the thickness of about 13 Å forms on the surface of anionic mica (Maier et al., 2015; Rapp et al., 2016), which is a substantial molecular barrier for adhesion. Another obstacle is the difficulties in achieving high cohesiveness in water. On one hand, some adhesives that rely on volatilization of solvent, such as polyvinyl alcohol and vinyl acetate-based glues (White and Wilker, 2011; North et al., 2017), are unable to cure underwater. On the other hand, water may weaken the cured structure of adhesives through hydrolytic degradation, plasticization or swelling over time (Narayanan et al., 2021). For example, it has been reported that the cured structures of some epoxy and polyurethane glues are prone to being broken by water molecules, leading to the decrease of adhesive strength over time (Li et al., 2018; Lee et al., 2020). All of these cases impede the long-term underwater adhesive strength.

## Strategies to remove interfacial water

**FIGURE 2:**
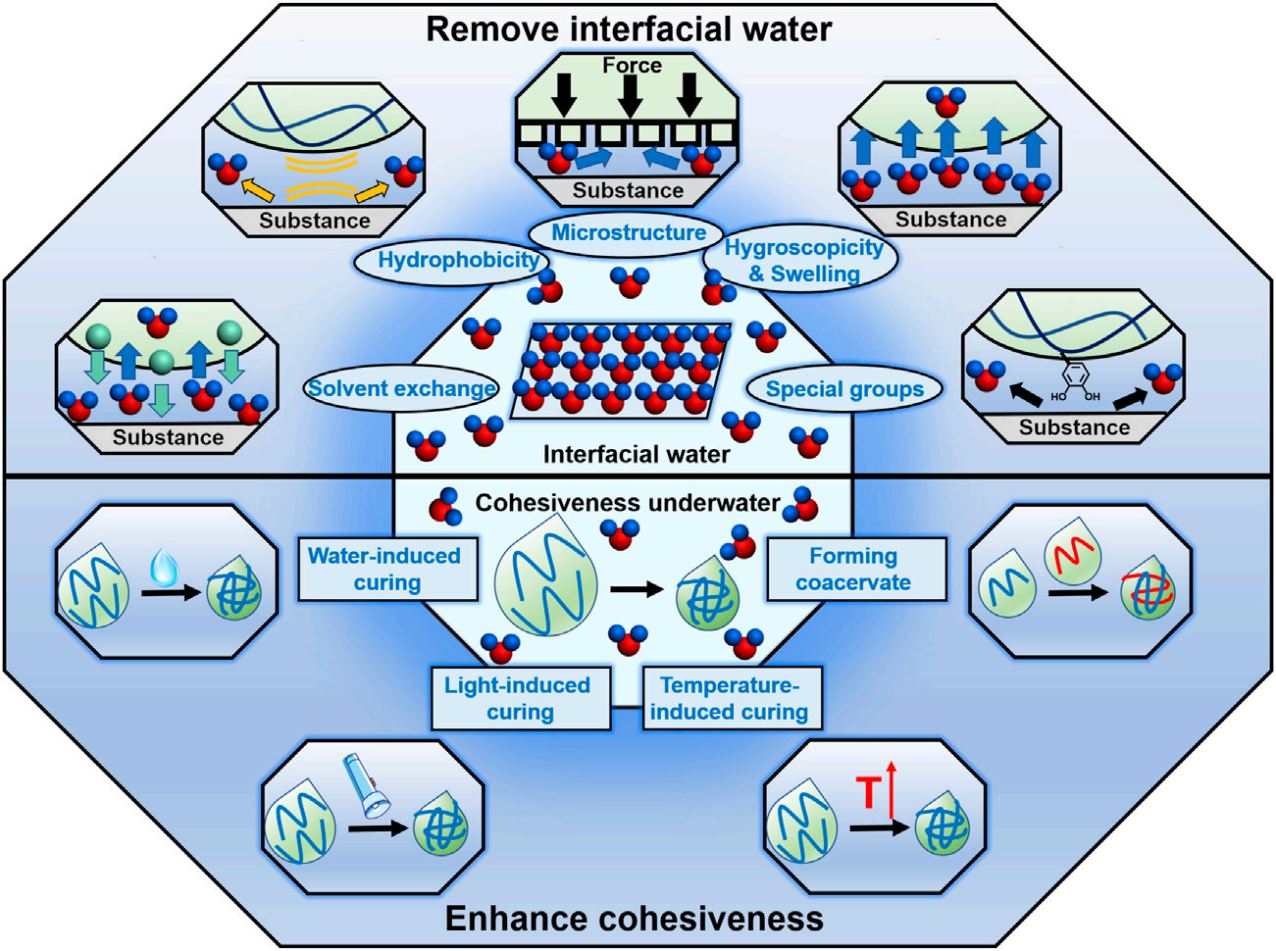
Strategies of removing interfacial water and enhancing cohesiveness underwater. Utilization of the hydrophobicity, hygroscopicity and swelling property, special groups of the adhesives, solvent exchange and bioinspired microstructures are the mainly reported strategies to remove the interfacial water. And there are also many strategies to enhance the cohesiveness of the underwater adhesives, including water-induced curing, light-induced curing, temperature-induced curing and forming coacervate.

Removing interfacial water is the essential step in underwater bonding. In recent years, researchers have proposed various strategies to remove interfacial water, and achieved macroscopical underwater adhesion (Figure 2). In the following, we systematically overviewed these elaborate strategies.

## Hydrophobicity

Hydrophobic interactions have been recently used in the design of underwater adhesives to overcome the barrier of interfacial water (Xu et al., 2017; Kaur et al., 2018; Cui et al., 2019; Wang Z. M. et al., 2021; Wu Y. C. et al., 2022; Liu et al., 2022). The hydrophobic segments were introduced into the adhesives through multiple reactions, such as Michael addition (Cui et al., 2019), polyesterification (Xu et al., 2017; Wang Z. M. et al., 2021), and free radical polymerization (Wu Y. C. et al., 2022). A representative example is the hyperbranched polymer (HBP) universal adhesive developed by Liu and coworkers (Cui et al., 2019). HBP with a hydrophobic backbone and hydrophilic catechol side branches was synthesized through Michael addition between multi-vinyl monomers and dopamine, and exhibited underwater adhesion performance. The authors proposed that upon contacting water, the hydrophobic segments were able to aggregate, thereby displacing the interfacial water molecules. Other typical examples were showed by Dhinojwala and coworkers who designed polyesters-based adhesives with hydrophobic aliphatic pendant groups (Xu et al., 2017; Kaur et al., 2018). In their study, the hydrophobic aliphatic chains were proposed to remove the bound water from the interface. In addition, directly utilizing the inner hydrophobic characteristic of natural polymer was another way to remove interfacial water. For example, Wang and coworkers reported a natural sericin protein-based self-hydrophobized adhesive (rSer-TA), whose hydrophobic chains were exposed by tris(2-carboxyethyl) phosphine (TCEP) reduction. It was proposed that after the adhesive was exposed to water, the hydrophobic chains can self-aggregate and therefore repel the interfacial water, after which the underwater interfacial adhesion was enhanced by hydrogen bonding or electrostatic interactions provided by catechol (Liu et al., 2022).

## Hygroscopicity and swelling property

Some underwater adhesives are inherent hydrophilic, and can remove the interfacial water (Pan et al., 2020; Wang et al., 2020). For example, Wang and coworkers developed an anthracenyl-functionalized polyethylenimine (anth-PEI) based adhesive, whose main polymer component PEI was hydrophilic. After the adhesive was applied underwater, it removed the interfacial water, benefiting for the intimate contact between the adhesive and the submerged surfaces (Wang et al., 2020). In addition, the swelling properties of hydrogels matrix were used to remove the interfacial water as reported by Zhang and coworkers (Pan et al., 2020). In their work, inorganic Ca_4_(AlO_2_)_6_SO_3_ -filled poly(acrylic amide) precursor solution was utilized as an adhesive. The authors proposed that after the adhesive was injected onto the substance underwater, the *in situ* formed hydrophilic poly(acrylic amide)-based matrix swelled and thus removed the interfacial water.

## Solvent exchange

The solvent exchange concept in underwater adhesion was developed by Waite and coworkers (Zhao et al., 2016). In this concept, the solvent of an adhesive, dimethylsulfoxide (DMSO), for example, was miscible with water. When the adhesive was extruded into water, the solvent exchange happened. As a result, the interfacial water entered into the adhesive, and the adhesive therefore well contacted with the submerged substance. This strategy was also illustrated by Dan and coworkers, who also utilized DMSO as the solvent (Song et al., 2021).

## Special functional groups

The functional groups of adhesives have also been reported to contribute to overcoming the barriers of interfacial water. A representative group was catechol, the special functional group that worked excellently in marine mussel’s adhesion (Lee et al., 2011; Hofman et al., 2018; Li S. D. et al., 2020). Due to its multiple interactions with various substance such as H bonds, covalent bonds, coordination interaction and π-π stacking (Moulay, 2014; Saiz-Poseu et al., 2019), catechol group has been introduced into the design of man-made adhesives through polymerization (Matos-Perez et al., 2012; Meredith et al., 2014; North et al., 2017; Tang et al., 2021), coupling reaction (Ryu et al., 2015; Shin et al., 2015; Li S. D. et al., 2020), Michael addition (Zhang et al., 2014; Cui et al., 2019), *in vivo* residue-specific incorporation strategy (Yang et al., 2014), etc. Even catechol group endowed the adhesives with considerable adhesive performance, only a few catechol-based adhesives that exhibited excellent underwater adhesion properties were reported (North et al., 2017; Zhan et al., 2017). A famous example was the poly(catechol-styrene) adhesive which was developed by Wilker and coworkers (North et al., 2017). This adhesive can strongly bond aluminum (Al) substance underwater with the maximal adhesive strength about 3 MPa. It was proposed that the surface water was broken by the “drilling down” properties of the incorporated catechol groups.

Another functional group is the isocyanate. This group is highly reactive, and widely used in the synthesis of polyurethane (PU) materials (Tseng et al., 2021; Xia et al., 2021; Yan et al., 2022). Due to the high reactivity, the isocyanate-containing adhesives can react with the interfacial water, and then form multiple bonds or interactions with the underwater substance, achieving strong underwater adhesion. For example, Huang and coworkers developed a hexamethylene diisocyanate-incorporated, polydimethylsiloxane-based adhesive (HDI-PDMS) by the addition reaction (Yan et al., 2022). It was proposed that the surface water molecules were removed by the reaction with the residual isocyanate groups in the adhesive. The underwater adhesion of isocyanate-containing adhesives was also verified by Wan and coworkers (Xia et al., 2021). In their work, the adhesive was synthesized by the reaction between tolylene diisocyanate (TDI) and diol, and the TDI was a little excessive to ensure the existence of diisocyanate in the adhesives. The results indicated that the adhesive exhibited strong adhesive properties to various submerged surfaces, including glass, Al, stainless steel (SS), et al.

## Structural underwater adhesives

Many natural organisms exhibited unique underwater adhesive performance. In recent years, learning from nature, researchers have designed numerous bioinspired structural underwater adhesives that are also capable of removing the interfacial water (Lee et al., 2007; Iturri et al., 2015; Baik et al., 2017; Ma et al., 2018; Yi et al., 2018; Lee et al., 2019; Wang and Hensel, 2021; Cui and Liu, 2021). For example, Pang and coworkers developed an octopus-inspired adhesive patch which contains dome-like protuberances that are similar to the suction cups of octopuses. It was proposed that after applying pressure, the interfacial water was removed into the upper chambers of the adhesives, and the pressure difference thus generated for underwater adhesion (Baik et al., 2017). Similarly, Kwak and coworkers developed a remora-inspired adhesive (RIA) that contains microstructures similar to the suction disk of remoras (Lee et al., 2019). The pressure difference was also produced by deformation of the adhesive after external forces, and the underwater adhesion was therefore achieved. In addition, other creatures, such as torrent frog (Iturri et al., 2015), mussels and geckos (Lee et al., 2007; Ma et al., 2018) also inspired researchers to design structural underwater adhesives recently. Compared with other adhesives, the structural underwater adhesives usually required elaborate structure design and complex fabrication process (Fan and Gong, 2021).

## Strategies of enhancing cohesion of underwater adhesives

In addition to the high interfacial adhesion, achieving macroscopical underwater bonding also requires the adhesives to possess high cohesion strength. Nowadays, researchers have developed numerous strategies to enhance the cohesiveness of underwater adhesives (Figure 2).

## Water-induced underwater curing

The adhesives that cure directly when encountering with water are convenient and easy-to-use in practical applications. Nowadays, various methods have been proposed to design this type of adhesives. A relatively simple method is to use facile chemical reactions to crosslink the adhesive. The solidification of epoxy-based adhesives is in this type. Typically, epoxy and curing agents, Mannich base, diamine for example (Choi et al., 2013; Zhou et al., 2018), are mixed before they are applied underwater, and then the mixture is able to cure underwater due to the crosslinking between epoxy groups and curing agents.

In addition, the curing process can be initiated when the adhesive encounters with water. There are several ways to achieve this type of curing, such as hydrophobicity-induced aggregation (Cui et al., 2019), water-incorporated chain extension reactions (Xia et al., 2021; Yan et al., 2022), solvent exchange-induced electrostatic complexation or hydrogen bond (H bond) crosslinking (Zhao et al., 2016; Song et al., 2021), etc. For example, the core of hydrophobic pentaerythritol tetraacrylate (PETEA) in a hyperbranched polymer with hydrophilic branches can self-aggregate upon encountering water (Cui et al., 2019), and the residual isocyanate in adhesive can react with water, thus extending chains and enhancing cohesiveness (Xia et al., 2021; Yan et al., 2022). The polyanion and polycation-mixed adhesive with the solvent of DMSO can solidify after it was applied underwater due to the production of electrostatic complexation caused by the exchange of DMSO and water (Zhao et al., 2016; Song et al., 2021). Besides, water can also be utilized to accelerate the curing process. The nanocomposite hydrogel-based adhesive developed by Zhang and coworkers was an example (Pan et al., 2020). In their work, the inorganic filters-incorporated hydrogel precursor slurry with initiator was used as an adhesive. After it was applied underwater, the inorganic Ca_4_(AlO_2_)_6_SO_3_ absorbed water, and then released heat, which further accelerated the free radical polymerization process.

## Light or temperature induced underwater curing

When exposed to light, anthracene undergoes dimerization reaction (Claus et al., 2017; Truong et al., 2017; Van Damme and Du Prez, 2018), thus forming crosslinking structures. Therefore, the adhesive polymer chains that contain anthracene can proceed photo-induced underwater crosslinking. One impressive example was the anthracenyl-functionalized polyethylenimine (anth-PEI)-based adhesives developed by Wang and coworker (Wang et al., 2020). In their work, LED blue light (λ > 400 nm) was utilized as the light source, and the curing of the anth-PEI adhesive happened within 2 min underwater, as shown by the fast increase of adhesive strength. In addition, the alkoxyphenacyl group or coumarin-contained materials can also be crosslinked upon light irradiation as illustrated by Joy and coworkers (Xu et al., 2017; Kaur et al., 2018; Tseng et al., 2021). For example, they developed alkoxyphenacyl-based polyurethane adhesives, and the changes in T_g_, rheology behaviors, and the peel strength all demonstrated the occurrence of crosslinking reactions after UV irradiating (Tseng et al., 2021).

The temperature change of an adhesive was also utilized to cure the adhesive. For example, Dong and coworkers reported a type of supramolecular adhesive (P1) from low-molecular-weight monomer that is formed by incorporating dibenzo-24-crown-8 to four-armed pentaerythritol. P1 exhibited a relatively low meting point (52°C), and can cure underwater by heating-cooling process (Li X. et al., 2020).

## Forming coacervate

Coacervate is a dense, highly concentrated, relatively low-viscosity, water-immiscible fluid with low interfacial energy, which has been reported to possess potentials to wet target surfaces and contribute to the following adhesion (Kaur et al., 2011; Wei et al., 2014). Forming coacervate was also an effective way to enhance the cohesiveness due to the generation of multiple interactions inside the adhesives in the coacervate formation process (Lee et al., 2020; Peng et al., 2020; Peng et al., 2021). Some coacervate-based adhesives, even showed no obvious liquid-solid transition underwater, can exhibit underwater adhesive capacities. The widely reported coacervate adhesive was formed through electrostatic interactions (Vahdati et al., 2020), as the sandcastle worms mainly use this interaction to produce glues (Shao and Stewart, 2010; Stewart et al., 2011a; Stewart et al., 2011b; Zhang et al., 2016). In addition, H bond can also be used to form coacervate-based underwater adhesives. For example, Lee and coworkers reported a coacervate adhesive, namely VATA, which is formed by mixing the poly(vinyl alcohol) (PVA) polymer and tannin (TA). The H bonds between the PVA and TA enhance the cohesiveness of the adhesive, achieving the macroscopical underwater bonding, though the adhesive did not cure underwater (Lee et al., 2020). Similarly, Zeng and coworkers directly mixed polyethylene glycol (PEG) and silicotungstic acid (SiW), and developed the SiW-PEG coacervate-based adhesive through H bonds. This coacervate adhesive was able to bond various wet surfaces, and treat bleeding (Peng et al., 2020). Through the combination of multiple interactions, the coacervate adhesives can also be formed. For example, Zeng and coworkers utilized hydrophobic interactions within poly(ethylene glycol)_77_-b-poly(propylene glycol)_29_-b-poly(ethylene glycol)_77_ (F68) and the H bonds between F68 and tannin forming a coacervate adhesive. It was able to exhibit underwater bonding capacities under the circumstances of coacervate state, even without curing (Peng et al., 2021).

Some types of coacervate-based adhesives can cure underwater. Commonly, the changes of water environment, such as temperature or pH, can induce curing (Shao and Stewart, 2010; Dompe et al., 2019). For example, Stewart and coworkers reported a sandcastle worms-mimetic coacervate-based adhesive consisting of aminated collagen, phosphodopa copolymer and some ions (Ca^2+^ and Mg^2+^). At a specific ions and polymer ratio, tuning the pH or increasing the temperature leads to curing of the coacervate (Shao and Stewart, 2010). Another example was reported by Kamperman and coworkers (Dompe et al., 2019). In their work, a complex coacervate based adhesive that consists of poly(N-isopropylacrylamide) (PNIPAM)-grafted oppositely charged polyelectrolytes was constructed. When the temperature of water environment was elevated higher than the lower critical solution temperature of PNIPAM, this adhesive can transform to non-flowing hydrogels underwater. In addition, the covalent crosslinking that happened within the coacervate, such as the oxidation of catechol groups-induced crosslinking (Shao and Stewart, 2010), azentidinium-induced crosslinking in base condition or over long time (Wei et al., 2019; Zhu et al., 2019; Wang Z. et al., 2021) and [2 + 2] cycloaddition reaction of coumarincan-induced crosslinking (Narayanan et al., 2020) also result in the curing of the coacervate-based adhesives. For these coacervate-based adhesives, the underwater adhesive strength was often higher than those of uncured coacervate-based adhesive, probably due to their stronger cohesiveness.

## Conclusion and perspectives

Realizing efficient underwater bonding is challenging due to the obstacles of interfacial water and difficulties in enhancing cohesiveness underwater. In this review, we summarized and overviewed the proposed strategies for overcoming these obstacles and difficulties. Despite that much progress has been made in underwater bonding, current underwater adhesives still have much space to made from the view of practical use. First, many adhesives require complex synthesis process or rigorous synthesis conditions (Pan et al., 2020). Although some adhesives showed high underwater bonding performance, the large production is also urgently needed (Li et al., 2022) for the commercialization. Second, the storability and usability of the adhesives should be carefully considered. Finally, current adhesives mainly focus on the underwater adhesive performance in static water environment, but the practical waters, such as lakes, rivers, and ocean are mainly dynamic. In dynamic water, the diffusion of the adhesive molecules accelerates, and the efficient bonding is more difficult to realize. Future works can focus on the dynamic environment of the water, thus developing underwater adhesives suitable for practical dynamic water environment.
